# Effects of modified multimodal analgesia on postoperative pain, sedation, and prognosis of gynecological patients

**DOI:** 10.1002/ibra.12002

**Published:** 2021-12-08

**Authors:** Fan Zhang, Man Luo, De‐Xing Liu, Yu‐Hang Zhu, Zhao‐Qiong Zhu

**Affiliations:** ^1^ Department of Anesthesiology Affiliated Hospital of Zunyi Medical University Zunyi Guizhou China; ^2^ College of Animal Science/Institute of Agro‐Bioengineering and Key Laboratory of Plant Resource Conservative and Germplam Innovation in Mountainous Region Guizhou University Guiyang Guizhou China

**Keywords:** gynecological surgery, pain relief, PONV, sedation, TAPB

## Abstract

Patient‐controlled intravenous analgesia is one of the most common pain relief methods in  the postoperative period, but its adverse reactions remain high. This study aimed to explore the role of improved combined analgesia methods in pain, sedation, postoperative nausea, and vomiting (PONV) in patients undergoing gynecological surgeries. This study was a prospective, randomized, double‐blind controlled study. A study population of 72 patients undergoing gynecological surgery were randomly assigned to either the TAPB + S group or the TAPB + N group. All patients in both groups underwent a transversus abdominis plane block (TAPB) after induction of anesthesia. The TAPB + S group received a continuous intravenous infusion (2 ml/h) of sufentanil (1 μg/kg) plus metoclopramide (30 mg) through 100 ml elastomeric pumps postoperatively. The TAPB + N group received a continuous intravenous infusion (2 ml/h) of nalbuphine hydrochloride (1 mg/kg) plus metoclopramide (30 mg) postoperatively. The main outcome measures were as follows: postoperative pain intensity, Ramsay sedation score (RSS) after surgery, PONV occurrence rate, and rescue analgesics. The RSS of the TAPB + S group was significantly higher than that of the TAPB + N group at 2, 4, and 6 h after the operation. However, the visual analog scale score of the TAPB + S group was much higher than that of the TAPB + N group. No significant differences were found between the two groups in terms of consumption of opioids and other narcotic drugs at 2, 4, 6, 24, and 48 h after the operation. No statistically significant differences were found with respect to PONV and other adverse events in both groups. Taken together, our data indicate that the TAPB + N program can provide better postoperative analgesia and also reduce the use of strong opioids. The more optimized scheme of perioperative analgesia still needs to be researched further.

## Graphical abstract

In this study, a randomized‐controlled clinical study was carried out to evaluate the effects of transversus abdominis plane block combined with opioid‐reducing anesthesia and analgesia on the prognosis of gynecological patients with the aim of optimizing clinical anesthesia management.

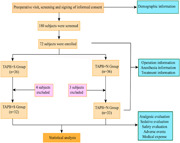

## 1 INTRODUCTION

Laparoscopic operation is favored by modern medicine because of its advantages of less trauma and rapid recovery. However, the incidence of postoperative complications including postoperative nausea and vomiting (PONV) can be as high as 80%, which may markedly affect the postoperative rehabilitation of patients.^1^ Female patients are more sensitive to pain; nerve block is rarely used in traditional gynecological anesthesia for technical reasons or equipment shortage. Opioids have always been the most commonly used analgesic drugs in general anesthesia, but the drug‐related complications are significant. In recent years, a series of optimization measures have been applied for the perioperative anesthesia and analgesia management of gynecological surgery to reduce the incidence of postoperative complications. Previous studies suggest that compared with combined intravenous and inhaled anesthesia, total intravenous anesthesia can reduce the incidence of PONV, and nerve block may further reduce the incidence of the above complications.^2^ The present study further explored the transversus abdominis plane block (TAPB) combined with opioid‐reducing patient‐controlled intravenous analgesia (PCIA) on sedation, pain, and PONV after gynecological laparoscopic operations. It is hoped that the multimodal analgesic scheme can maximize drug efficacy and minimize side effects, which can be used to guide clinical work and improve medical quality and patient experiences in the future.

## 2 MATERIALS AND METHODS

### 2.1 Patient selection

The institutional ethics committee of Zunyi Medical University (Guizhou, China) approved this study [no.2019(4)]. Written informed consents were obtained from all subjects. This study has been registered at chictr.org.cn (no.ChiCTR100021408) before initiation. Patients hospitalized from June 2020 to December 2020 were included in this clinical study chronologically. All potential participants were screened first by the research stuff; we screened 180 patients who had been planned for elective gynecological surgery on the basis of the inclusion and exclusion criteria.

The inclusion criteria were as follows: patients undergoing elective laparoscopic gynecological surgery, between 18 and 65 years of age, body mass index (BMI) 18–28 kg/m^2^, American Society of Anesthesiologists (ASA) physical status Ⅰ–Ⅱ, and estimated operation time within 3 h. The exclusion criteria were as follows: severe systemic diseases requiring ICU treatment after operation, pregnancy and lactation, abnormal coagulation function, a history of use of antiemetic drugs within 24 h, operation time more than 3 h, or presence of scars, infections, tumors, and so forth, on the puncture site. The elimination criteria were as follows: conversion to open surgery, perioperative use of antiemetics and analgesics other than the study protocol, poor compliance, and missing data. Also, subjects could withdraw from the study for any reason.

### 2.2 Anesthesia induction

After entering the operating room and completing the baseline data recording, all patients included in this study were randomly divided into the TAPB + S group and the TAPB + N group according to the random number table methods. All patients received anesthesia induction in a uniform manner: midazolam (20180505, Enhua) 0.1 mg/kg, sufentanil (1180206, Yichang Humanwell) 0.3 μg/kg, etomidate (1806263, Guorui) 0.3 mg/kg, and rocuronium (180403, Xianju) 0.6 mg/kg. After endotracheal intubation, the parameters of the anesthesia machines were set for mechanical ventilation: tidal volume 6–8 ml/kg, respiratory rate 12–16 times/min, and PetCO_2_ maintained within 35–45 mmHg. Propofol (1806263, Guorui) 4–10 mg/kg/h and remifentanil (6180411, Yichang Humanwell) 0.25–4 μg/kg/min were injected intravenously to maintain anesthesia. All patients were maintained with total intravenous anesthesia, and general anesthesia maintenance drugs were stopped at the end of the operation.

### 2.3 TAPB treatment process

All patients underwent TAPB treatment after anesthesia induction, which was completed by the same anesthesiologist who was proficient in administering ultrasound‐guided nerve blocks. Patients were placed in the supine position after the induction of general anesthesia. After routine disinfection and towel‐laying preparation, a 6–12 MHz high‐frequency linear array probe of a portable color Doppler ultrasound diagnostic instrument (M9, Mindray) was positioned under the costal margin. The probe scanned along the axillary front and moved to the ventrolateral side until it was perpendicular to the axillary midline. When the ultrasound clearly showed the three‐layer muscle structure of the external oblique muscle, internal oblique muscle, and transverse abdominal muscle, the position of the transverse abdominal fascia was determined by NS 0.5 ml injection and then replaced by 0.33% ropivacaine (03B1371, Yichang Humanwell) 15 ml on each side to achieve analgesia effects.

### 2.4 Postoperative analgesia scheme and pain remedy

The drug interventions agreed to all participants in the study were as follows: At the end of the operation, all patients were administered metoclopramide (62009041, Suicheng) 30 mg intravenously for advanced PONV prevention. Then, the patients were connected to a PCIA pump (WZ‐6523C4, Royal Fornia Medical Equipment). The TAPB + S group received a continuous intravenous infusion (2 ml/h) of sufentanil (1180206, Yichang Humanwell) (1 μg/kg) plus metoclopramide (62009041, Suicheng) (30 mg) through 100 ml elastomeric pumps postoperatively. The TAPB + N group received a continuous intravenous infusion (2 ml/h) of nalbuphine hydrochloride (11j03081, Yichang Humanwell) (1 mg/kg) plus metoclopramide (62009041, Suicheng) (30 mg) through 100 ml elastomeric pumps postoperatively. The PCIA parameters were as follows: total amount 100 ml, background infusion rate 2 ml/h, and bolus dose 0.5 ml, with a lock‐out of 15 min. The unlabeled study drug was configured by an anesthesia nurse who was not involved in this trial and who provided the drug to clinical anesthesia practitioners. If the pain intensity was >4 on a 10 cm visual analog scale (VAS), a single dose of paresibuna (F2102032, Aosaikang Pharm) 40 mg was administered for pain relief; the time interval was 6 h. If there was no remission during this period of time, sufentanil (1180206, Yichang Humanwell) 5 μg was added to relieve pain again. No special treatment was required for Grade 1–2 PONV, and  the changes in the condition were closely observed. Patients with Grade 3 PONV were administered metoclopramide (62009041) 10 mg intravenously for the first time, and the patients without remission or those with Grade 4 PONV were administered ondansetron (19090918, Medco) 8 mg intravenously for the secondary remedial treatment.

### 2.5 Outcome measures

Our primary outcome measure was the postoperative pain intensity assessed after tracheal extubation and subsequently at 2, 4, 6, 24, and 48 h. The VAS scores were recorded by an anesthesiologist who was not aware of the type of treatment administered. The use of rescue analgesic drugs and any adverse effects were recorded carefully in the case report forms. The frequency, intensity, and remedial measures of PONV at each of the above time points after the operation were also recorded. The Ramsay sedation scale (RSS) was used to evaluate the degree of postoperative sedation for both groups. Our secondary outcomes included the incidence and mortality of complications, postanesthesia care unit (PACU) discharge time, postoperative drainage extraction time, length of hospital stay, and also medical expenses.

### 2.6 Statistical analysis

The sample size calculation was based on the primary outcome. According to previous research data, it was assumed that the 24 h VAS would be similar to that in previous studies. Using PASS 15.0 software (NCSS, LLC), based on a two‐sided test of the log‐rank test module with a power of 80%, a significance level of 0.05, and a 20% loss rate, the total sample size was calculated to be 72 (36 in each group).

For numeric variables, the Kolmogorov–Smirnov test was used to verify normality. Normally distributed variables are expressed as the mean (SD), and abnormally distributed variables are expressed using the median (interquartile range). Categorical variables are expressed as numbers (percentages). Independent two‐sample *t*‐tests were used to compare normally distributed variables. Abnormally distributed variables and ranked data were compared using the Mann–Whitney *U*‐test. Categorical variables were analyzed using the *χ* ^2^ test or Fisher's exact test. Data were analyzed using SPSS (version 24.0, SPSS Inc., IBM). All statistical tests were two‐tailed, and a *p* value <0.05 was considered statistically significant.

## 3 RESULTS

**Figure 1:**
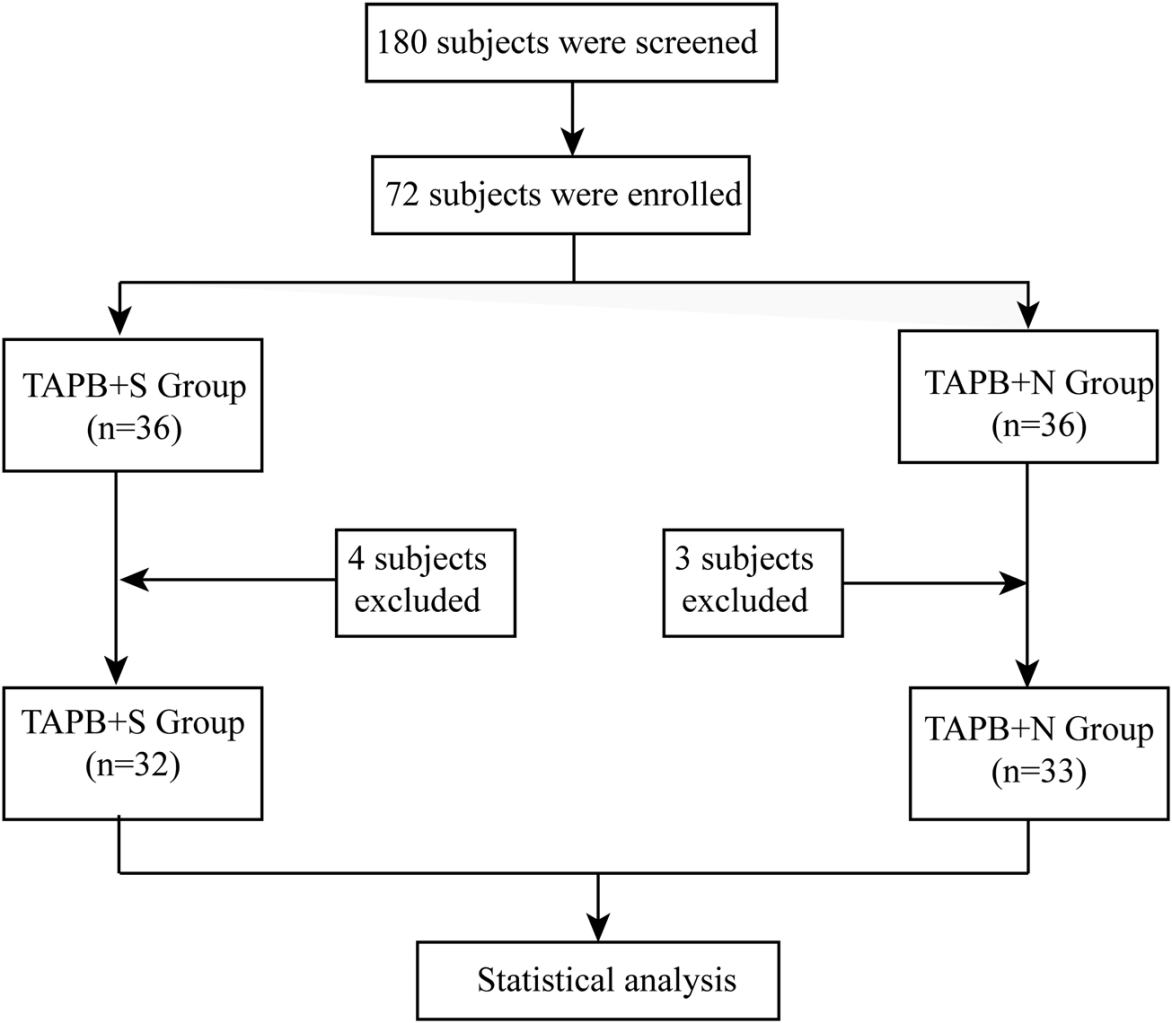
Study selection flow chart. TAPB, transversus abdominis plane block

A total of 72 patients were included and randomly assigned to the TAPB + S group or the TAPB + N group. During the follow‐up period, seven participants were excluded from the analysis because they violated the study protocol for various reasons (four participants in the TAPB + S group and three participants in the TAPB + N group). Finally, 32 patients who were included in the TAPB + S group and 33 patients who were included in the TAPB + N group entered the analysis stage. The complete flow chart of participant selection in this study is shown in Figure 1.

### 3.1 Demographic information, anesthesia, and surgical information

There were no significant differences between the TAPB + S group and the TAPB + N group in demographic characteristics such as age, height, weight, BMI, smoking history, history of PONV or dizziness, ASA classification, diagnosis, volume, anesthesia, and operation time. There was also no significant difference between the two groups in hemodynamics and the use of intraoperative anesthetics. The baseline indexes of the two groups were homogeneous (Table 1, *p* > 0.05).

**Table 1:** Comparison of basic data between the two groups

Baseline indicators	TAPB + S group (*n* = 32)	TAPB + N group (*n* = 33)	2/*t*	*p*
Age (year)	36.7 ± 10.2	36.4 ± 9.2	1.138	0.258
Height (cm)	158.3 ± 5.0	156.4 ± 4.0	0.556	0.580
Weight (kg)	55.7 ± 6.8	55.6 ± 5.9	0.782	0.436
BMI (kg/m^2^)	22.3 ± 2.5	22.7 ± 2.4	0.657	0.512
PONV history or motion sickness				
Yes	10 (31.3%)	9 (27.3%)	0.088	0.767
No	22 (68.7%)	24 (72.7%)	0.088	0.767
ASA classification				
Ⅰ	17 (53.1%)	20 (60.6%)	0.815	0.367
Ⅱ	15 (46.9%)	13 (39.4%)	0.815	0.367
Diagnosis				
Oophoritic cyst	20 (62.5%)	17 (51.5%)	0.226	0.635
Myoma of the uterus	4 (12.5%)	6 (18.2%)	0.633	0.426
Intima high‐grade lesions	4 (12.5%)	3 (9.1%)	0.050	0.824
Anesthesia time (min)	139.1 ± 43.7	146.1 ± 40.6	0.108	0.914
Operation time (min)	98.6 ± 39.5	100.9 ± 35.3	0.236	0.814
Intraoperative volume (ml)				
Liquid volume	1398.4 ± 267.5	1493.9 ± 306.4	0.847	0.399
Urine volume	392.2 ± 235.9	324.2 ± 208.8	0.314	0.754

Abbreviations: ASA, American Society of Anesthesiologists; BMI, body mass index; PONV, postoperative nausea and vomiting; TAPB, transversus abdominis plane block.

### 3.2 Evaluation of analgesic and sedative effects

**Figure 2:**
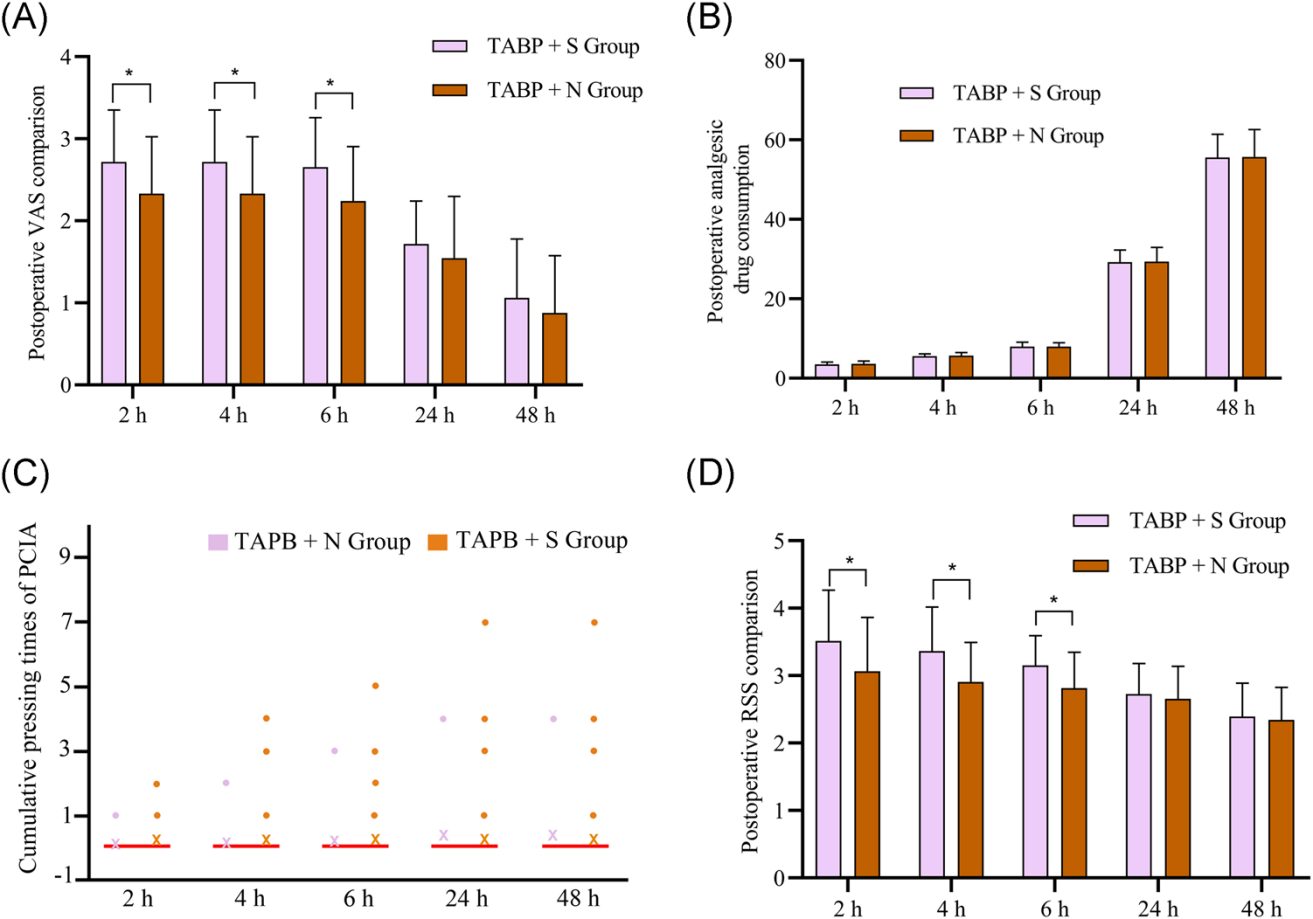
Comparison of visual analog scale (VAS) and Ramsay sedation scale (RSS) between the two groups. (A) Postoperative VAS of both groups. (B) Postoperative analgesic drug consumption. (C) Cumulative pressing times of the patient‐controlled intravenous analgesia (PCIA) pump. (D) Postoperative RSS comparison. The data are expressed as mean ± standard deviation. *p values < 0.05 [Color figure can be viewed at wileyonlinelibrary.com]

The VAS scores at 2, 4, and 6 h in the TAPB + S group were significantly higher than those in the TAPB + N group (Figure 2A, *p* < 0.05). There was no significant difference between groups in the consumption of sufentanil (μg)/nalbuphine (mg) at 2, 4, 6, 24, and 48 h after the operation, and the cumulative pressing times of PCIA pumps (Figure 2B,C, *p* > 0.05). Compared with the TAPB + N group, the RSS scores at 2, 4, and 6 h in the TAPB + S group were significantly higher; the depth of postoperative sedation was stronger in this group (Figure 2D, *p* > 0.05).

### 3.3 Evaluation of PONV control effects

**Figure 3:**
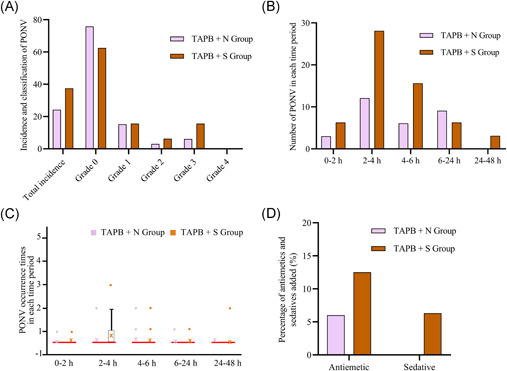
Comparison of postoperative nausea and vomiting (PONV) between the two groups. *(*A) Total incidence and classification of PONV. (B) Numbers of patients with PONV in each time period. (C) PONV occurrence times in each time period. (D) Postoperative PONV remedy. The data are expressed as mean ± standard deviation. *p* values > 0.05 [Color figure can be viewed at wileyonlinelibrary.com]

No occurrence of PONV was observed in both groups during the postoperative PACU observation period. There were no cases of Grade 4 PONV or above in both groups (Figure 3A, *p* > 0.05). Compared with the TAPB + N group, there was no significant difference in the incidence, frequency, or remedy drug consumption of PONV after the operation at each time point (Figure 3B–D, *p* > 0.05).

### 3.4 Comparison of secondary indicators

With regard to the secondary indicators, compared with the TAPB + N group, there was no significant difference in the incidence of opioids analgesic‐related complications or TAPB‐related complications, and no deaths were reported during this study. Hemodynamic indexes such as heart rate, mean arterial pressure, and SpO_2_ between the two groups at each time point were similar. There was no significant difference in the resuscitation time, PACU residence time, urinary catheter extraction time, drainage tube extraction time, total hospital stay, postoperative hospital stay, and medical expenses between the two groups (Table 2, *p* > 0.05).

**Table 2:** Comparison of prognostic indicators between the two groups

Outcome measures	TAPB + S (*n* = 32)	TAPB + N (*n* = 33)	*t*	*P*
Extubation time (min)	13.3 ± 9.8	12.6 ± 6.6	0.341	0.734
PACU time (min)	48.6 ± 9.7	48.3 ± 9.9	0.107	0.915
Catheter extraction time (h)	30.3 ± 10.8	27.8 ± 10.9	0.927	0.358
Drainage tube extraction time (h)	60.6 ± 12.8	58.4 ± 15.2	0.654	0.515
Total hospital stay (day)	8.6 ± 2.7	8.5 ± 2.1	0.234	0.816
Postoperative hospital stay (day)	4.9 ± 1.5	4.9 ± 1.1	−0.006	0.995
Medical expense (10,000 yuan)	1.4 ± 0.3	1.4 ± 0.2	0.244	0.808

## 4 DISCUSSION

In recent years, minimally invasive gynecological endoscopic surgery has developed into the main clinical operation type recommended. However, the incidences of anesthesia‐related complications such as nausea and vomiting, deep sedation, and respiratory depression after gynecological endoscopic surgery are still high. Although the above complications are usually not life‐threatening, they can still lead to dehydration, electrolyte disorders, and wound dehiscence, and also prolong hospital stay, and increase treatment costs.^3^, ^4^, ^5^ Enhanced recovery after surgery (ERAS) puts forward a series of optimization measures to accelerate the rehabilitation of surgical patients, such as anti‐stress and anti‐inflammatory to optimize anesthesia management of the surgical patients.

This study is part of a continuous series of studies designed to optimize the anesthesia and analgesia program for gynecological patients. In the previous study,^2^ we first confirmed that the incidence of PONV under total intravenous anesthesia was lower than that under combined intravenous and inhaled anesthesia. It has been reported that halogenated inhalation anesthetics increased the incidence of PONV compared with intravenous anesthetics, and the mechanism for this might be related to the expansion of the gastrointestinal tract, flatulence, and increase in cerebral blood volume.^6^ The intravenous anesthetic propofol has antinausea and vomiting effects, which may be the reason for the low incidence of PONV in the intravenous group in several studies.^7^ Second, we confirmed that TAPB combined with total intravenous anesthesia could eliminate the conventional intravenous analgesic load after gynecological laparoscopic surgery, which shortened the postoperative recovery time and reduced opioids consumption. To reduce the severe pain induced by anesthesia and surgical trauma after an operation, low‐dose intravenous analgesics were often used within half an hour before the end of the operation to combine the two stages of intraoperative anesthesia maintenance and postoperative analgesia. Our previous study showed that TAPB improved the analgesic effects of PCIA and effectively alleviated the postoperative pain of patients undergoing gynecological surgery. The load of intravenous analgesia commonly used in the clinic could be eliminated, so as to reduce the dosage of opioids. On the basis of our previous research, the purpose of the present study was to observe whether patients could further adopt less opioids analgesia scheme to optimize the anesthesia management.

In addition to the pain directly induced by the incision, the pain caused by CO_2_ pneumoperitoneum is more significant. Its mechanism is visceral pain caused by nerve traction trauma and inflammatory release after pneumoperitoneum, which is often accompanied by sweating, anxiety, irritability, and other symptoms.^8^, ^9^ PCIA is the most commonly used analgesic method after laparoscopic surgery. It has the advantages of simplicity, convenience, safety, and achievement of a relatively stable blood drug concentration. Patients can adjust the analgesic intensity according to different analgesic requirements, so as to meet the personalized analgesic needs to the greatest extent. Opioids have been the first‐choice analgesics for PCIA for many years in China. Sufentanil is widely used in the clinic, but its analgesic effect on visceral pain is poor. Although opioids are effective analgesics, they are also frequently associated with adverse effects such as nausea, sedation, constipation, and, in some cases, respiratory depression.^10^, ^11^, ^12^, ^13^

The regional anesthesia technique is an effective mode of analgesia, considered safe with a low risk for complications, and is a frequently utilized component in multimodal analgesic regimens. TAPB is a regional anesthesia technique that provides strong analgesia to the anterior abdominal wall. Hutchins et al.^14^, ^15^, ^16^ reported that TAPB provided good postoperative analgesic effects for up to 3 days. As a pain treatment method for abdominal surgery, TAPB can reduce the stress response of surgical trauma stimulation to the body, lead to decreased use of perioperative anesthetics, decrease postoperative‐related complications such as PONV, and accelerate the postoperative rehabilitation of patients.^17^, ^18^

PONV is the result of a variety of etiologies, stimuli, and receptors. Its specific pathogenesis is still unclear. Women are at a high risk of developing PONV. The influence of surgical factors on PONV mainly depends on the operation time and operation type. With every 30 min in operation, the risk of PONV increases by 60%. The risk factors related to anesthesia mainly include the use of volatile anesthetics and the use of opioids.^19^, ^20^ Studies have found that peripheral blood κ opioid receptors distributed in the visceral afferent nerves of the gastrointestinal tract are the key regulators of visceral pain. In addition, the incidence of respiratory inhibition of activating these receptors is low with a capping effect, which provides a new drug choice for postoperative analgesia in gynecological laparoscopic surgery.^21^, ^22^, ^23^ This study investigated the effects of TAPB combined with an optimized PCIA scheme with use of fewer opioids in gynecological laparoscopic surgery to provide good analgesia, reduce PONV, and provide patients with better medical services.

The results of this study showed that the VAS scores of the nalbuphine postoperative analgesia formula at 2, 4, and 6 h after the operation were better than that of sufentanil, suggesting that the analgesic effects of nalbuphine may be better than sufentanil after gynecological laparoscopic surgery. Studies have reported that nalbuphine excited κ receptors, decreased the release of neurotransmitters such as substance P and glutamate, and reduced the transmission of peripheral pain signals to the central nervous system to produce analgesic effects; it is worth mentioning that the analgesic effects of nalbuphine on visceral pain are more potent.^24^, ^25^, ^26^ Therefore, the results of this study suggest that nalbuphine has a stronger analgesic effect on female patients undergoing laparoscopic surgery, which may be related to the unique therapeutic effect of nalbuphine on visceral pain. It is reported in the literature^27^ that there were no significant differences in the analgesic effects between PCIA alone and the “PCIA + background infusion dose,” but the incidence of postoperative adverse events of the latter was significantly higher. Therefore, the ASA does not recommend setting the background dose. The results of this study showed that there were no significant differences in the VAS scores and postoperative sufentanil consumption within 48 h during the study periods, and the postoperative VAS pain score was mostly less than 3 in both groups. Therefore, while canceling the load, we can also consider canceling the background dose for further study.

The RSS scores of all patients at 2, 4, and 6 h after the operation were between 2 and 4, indicating that the postoperative sedation levels of the two postoperative analgesia schemes were appropriate. However, the RSS of patients with postoperative analgesia with nalborphine was significantly lower than that of the sufentanil group at 2, 4, and 6 h after the operation. It has been reported that low‐dose κ receptor agonists can produce anti‐anxiety effects and improve the sedation score, which may be one of the reasons why the postoperative sedation score of the TAPB + N group is lower than that of the TAPB + S group. Excessive sedation has major disadvantages; patients may have critical situations such as respiratory depression due to excessive sedation. Good analgesic and sedative effects can effectively eliminate patients' negative emotions such as anxiety and depression. Therefore, nalbuphine is recommended for postoperative analgesia in female patients after laparoscopic surgery.

The results of this study showed that the incidence of PONV decreased in the TAPB + N group, but it still occurred in both groups, and the difference was not obvious; therefore, the advantages of μ‐opioid receptor antagonism were not highlighted in this study. Studies have found that nalbuphine can inhibit the synthesis and release of excitatory mediators in the trigger region of emetic chemical receptors, but nalbuphine κ receptor agonists themselves rarely lead to PONV, and nalbuphine is only a weak μ receptor antagonist, which may be the reason why there is no significant difference in PONV between the two groups.^28^ From the results of this study, it is found that PONV is not directly related to visceral pain, but further research is required to  determine whether it is related to the hormone levels in patients. Considering that all subjects are women, this study did not detect estrogen.

It has been reported in the literature that nalbuphine is a new opioid receptor agonist–antagonist mixed analgesic, which can antagonize μ receptors to some extent, reducing μ‐receptor‐related side effects such as PONV. Theoretically, the incidence of adverse events (such as PONV, constipation, urinary retention, etc.) related to postoperative analgesia with nalbuphine is lower than that of sufentanil, but the results of this study found no significant difference between the two postoperative analgesia schemes. It can be seen that the postoperative analgesia regimen of nalbuphine failed to highlight μ‐opioid receptor antagonist advantages of reducing adverse events such as PONV, constipation, and urinary retention. In conclusion, nalbuphine has a good therapeutic effect on visceral pain in gynecological laparoscopic surgery, but it does not significantly improve the occurrence of PONV. Furthermore, during the course of this study, all patients had received TAPB treatment before; thus, the total dosage of intravenous analgesics decreased, which may also be the reason why there was no significant difference in the occurrence of PONV between the two groups. With an increase in the dosage of opioids, the side effects will further increase. This also reflects the advantages of improved multimodal analgesia.

In recent years, the anesthsiologists pay more and more attention to the standardized and rational application of opioids, and even advocate the treatment strategy of “de opioids” or “less opioids” in the perioperative period, but “de opioids” cannot be achieved under the existing medical conditions. In this study, it was found that PONV still occurred in patients undergoing gynecological laparoscopic surgery with less opioid postoperative analgesia. Therefore, in the follow‐up study, it is necessary to further explore the impact of opioid‐free perioperative analgesia on postoperative pain and PONV.

In summary, this study represents an exploration of the optimal scheme of perioperative analgesia and anesthesia for patients undergoing gynecological endoscopic surgery. TAPB was used as the basic analgesia before gynecological laparoscopic surgery. The postoperative opioid‐reducing analgesia scheme can provide good analgesia for patients, but PONV still occurs and further research is needed in the future.

## CONFLICT OF INTERESTS

The authors declare that there are no conflict of interests.

## ETHICAL STATEMENT

All the experiments on the involved participants were approved by the Ethics Committee of Biomedical Research for Affiliated Hospital of Zunyi Medical University [no. 2019(4)]. This study has been registered at chictr.org.cn (no. ChiCTR100021408) and was conducted following the Declaration of Helsinki.

## AUTHOR CONTRIBUTIONS

Fan Zhang contributed to the design, interpretation, and writing of the study. Man Luo contributed to implementation of the study. De‐Xing Liu contributed to participant follow‐up. Yu‐Hang Zhu contributed to data analysis, and Zhao‐Qiong Zhu contributed to clinical research coordination and quality control. All authors contributed to drafting and revising the article, gave final approval of the version to be published, and agree to be accountable for all aspects of the work.

## Data Availability

The data that support the findings of this study are available on request from the corresponding author. The data are not publicly available due to privacy or ethical restrictions.
